# Public Health Crisis and Health Policy Challenges in Iran: Safeguarding Civilian Health Amid Regional Instability

**DOI:** 10.34172/ijhpm.9865

**Published:** 2026-06-13

**Authors:** Fangwei Xue, Peilin Han

**Affiliations:** Shandong First Medical University, Jinan, China.

## Dear Editor,

 We are writing to focus on the significant public health challenges arising from the escalation of regional tensions in Iran and the associated global health policy issues. The recent attacks on civilian energy facilities in Tehran have further exacerbated the already strained public health situation in Iran, seriously undermining the fundamental right to health of civilians. Meanwhile, it has posed urgent and critical challenges to health policy formulation and health system resilience building in the context of conflicts.

 Prolonged regional tensions have placed considerable strain on Iran’s public health system. The sharp depreciation of the national currency—with the Iranian rial losing over 80% of its value between 2018 and 2024 under the weight of prolonged sanctions and economic isolation—has led to a surge in the prices of essential medical supplies, healthcare services and health-related daily necessities—with prices of imported medicines reported to have increased by over 300% and domestic pharmaceutical production severely hampered by restricted access to raw materials^2,3^—, resulting in a drastic decline in their accessibility. Millions of ordinary families are unable to obtain basic health security—data indicate that out-of-pocket health expenditure has reached unsustainable levels for a significant proportion of Iranian households, disproportionately affecting low-income groups.^4^ Vulnerable groups including children, the elderly and patients with chronic diseases have become the primary affected population, with health equity continuing to deteriorate. The service supply capacity and emergency response capacity of Iran’s health system have been significantly constrained due to resource scarcity, making it difficult to advance the basic goal of universal health coverage.^1,4^

 The recent escalation of regional tensions has compounded Iran’s already strained public health landscape. The overstretched health system now faces unprecedented additional pressure: surging demand for injury, trauma, and conflict-related care; disruption of routine healthcare services; deteriorating operational capacity of health facilities; and the loss of effective support for both immediate and long-term civilian health needs.^1,4^ Evidence from broader conflict settings—drawn from cross-national analyses—documents critical shortages of surgical supplies and blood products, declines in routine immunization coverage and maternal care attendance, and marked deterioration of mental health indicators among conflict-affected populations^5^—patterns to which Iran’s sanctions-weakened health system is particularly susceptible. This situation underscores the direct impact of health system vulnerability on population health in conflict contexts, and highlights the critical role of evidence-based health policies in safeguarding civilian health during acute crises.

 From the professional perspective of global health policy and management, safeguarding civilian health in conflict scenarios is the core embodiment of humanitarian principles and an important issue of global health governance. Regardless of the evolution of regional tensions, ensuring the accessibility of basic healthcare services, maintaining the basic operation of the health system and protecting the health rights and interests of vulnerable groups should be an unshakable policy bottom line. To this end, we propose the following specific and actionable policy recommendations: (*i*) immediate implementation of humanitarian exemptions for the import and distribution of essential medicines, medical devices, and health commodities to restore disrupted supply chains; (*ii*) establishment of an internationally coordinated emergency health fund under World Health Organization (WHO) leadership to sustain primary healthcare facilities in conflict-affected areas; (*iii*) prioritization of maternal and child health services, chronic disease management, and mental health support programs, which evidence shows are disproportionately disrupted in conflict settings; and (*iv*) integration of health system resilience indicators into post-conflict reconstruction frameworks to prevent long-term collapse of health infrastructure. All countries and the international community must uphold the principles of health equity and global health security, and take concrete action to prevent civilians from suffering further health damage due to non-health factors.^1-5^

 As researchers in the field of health policy and management, we emphasize that the protection of public health in the context of conflicts is in urgent need of support from scientific and pragmatic health policies, as well as the coordinated actions of the international community. We call on all parties to abide by humanitarian norms, put the safeguarding of civilian health in the first place, and take practical measures to address Iran’s public health challenges and ensure the sustainable supply of basic healthcare services. We also advocate the peaceful settlement of regional disputes to eliminate the external factors affecting public health security at the source. We sincerely hope that this letter can arouse in-depth attention from the academic community and policy-makers to public health issues in conflict-affected regions, promote more empirical health policy research and practice, strengthen the capacity of the global health governance system to protect civilian health in conflict-affected regions, and safeguard the fundamental health rights of all human beings.

## Disclosure of artificial intelligence (AI) use

 Not applicable.

## Ethical issues

 Not applicable.

## Conflicts of interest

 Authors declare that they have no conflicts of interest.

## Disclaimer

 This article represents the personal views of the author only and is not associated with the journal or the author’s institution.
